# Nodular Lymphocyte Predominant Hodgkin Lymphoma of the Ileum

**DOI:** 10.1155/2017/5981013

**Published:** 2017-11-02

**Authors:** Aruna Rangan, Sarah W. Grahn, Andrew L. Feldman

**Affiliations:** ^1^Department of Laboratory Medicine and Pathology, Mayo Clinic, Rochester, MN, USA; ^2^Colon and Rectal Surgery Associates, Burnsville, MN, USA

## Abstract

Nodular lymphocyte predominant Hodgkin lymphoma (NLPHL) is a rare lymphoma derived from germinal center B lymphocytes that typically presents with localized lymph node involvement and can mimic a variety of both reactive and other neoplastic conditions. Extranodal involvement is uncommon in NLPHL and typically occurs in the context of previously documented or synchronous nodal disease. Involvement of the gastrointestinal tract is exceedingly rare. Here, we present the first case to our knowledge of NLPHL involving the ileum that was discovered incidentally on routine screening colonoscopy in an asymptomatic patient. An awareness of the spectrum of clinical presentations, careful morphologic evaluation, and a comprehensive panel of immunohistochemical stains are essential for correct diagnosis of NLPHL presenting in unusual anatomic sites.

## 1. Introduction

Nodular lymphocyte predominant Hodgkin lymphoma (NLPHL) is a rare lymphoma derived from germinal center B lymphocytes that represents about 5% of all Hodgkin lymphomas in Western countries [1]. It is characterized morphologically by a nodular infiltrate containing scattered large neoplastic “lymphocyte predominant” (LP) cells in a background of small lymphocytes, histiocytes, and expanded follicular dendritic cell meshworks. Most patients present clinically with localized peripheral lymphadenopathy, particularly involving cervical or axillary lymph nodes or occasionally inguinal or femoral nodes. Extranodal involvement by NLPHL is rare and usually arises in the context of documented nodal disease and/or in other lymphoreticular organs. Involvement of the gastrointestinal tract is extremely rare. Here, we report what is to our knowledge the first case of NLPHL identified in the ileum as an incidental finding detected on colonoscopy in an asymptomatic patient.

## 2. Case Report/Pathologic Findings

**Figure 1:**
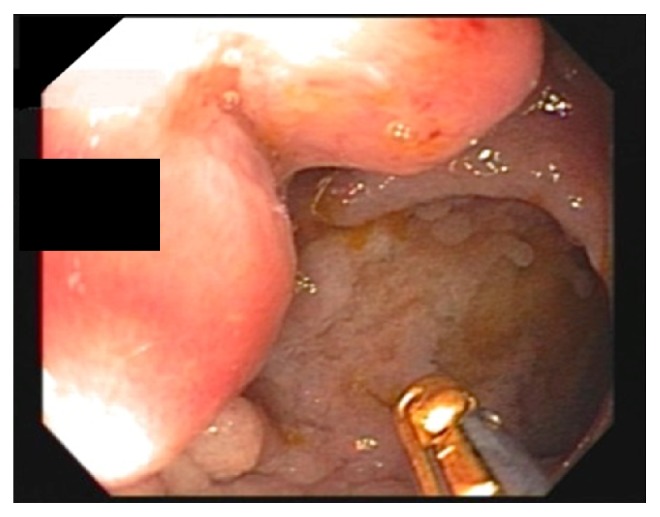
Endoscopic image of ulcerated polypoid lesion discovered in the terminal ileum during routine screening colonoscopy.

A 54-year-old asymptomatic male underwent routine screening colonoscopy. A 2.0 cm polypoid, nonobstructing, ulcerated lesion was seen just inside the ileocecal valve (Figure 1). A 1.0 cm portion of the polyp was removed with a hot snare and submitted for pathologic evaluation.

**Figure 2:**
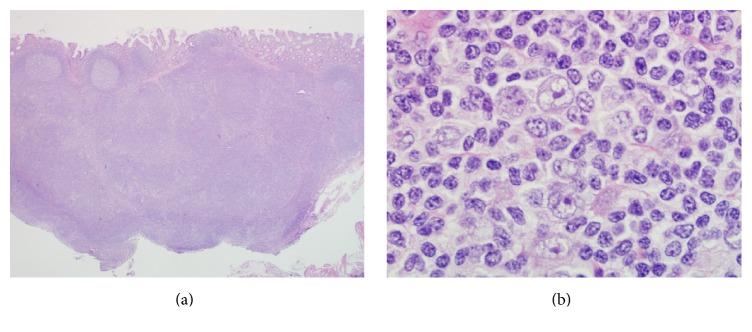
Morphologic features of the ileal lesion. (a) Low-power image of an H&E stain shows a vaguely nodular infiltrate with several adjacent reactive secondary lymphoid follicles. (b) High-power image of an H&E stain shows scattered large cells with multilobated nuclei (lymphocyte predominant cells) in a background of small lymphocytes and occasional histiocytes.

Four micron thick sections were cut from paraffin blocks of the terminal ileum and stained with hematoxylin and eosin (H&E) and immunostains for CD20, CD3, CD30, CD15, CD45, CD21, EMA, BCL6, OCT2, PAX5, IgD, PD-1, and kappa and lambda immunoglobulin light chains. Sections from the ileal lesion showed expansion of the submucosa by a lymphohistiocytic infiltrate with a vaguely nodular pattern (Figure 2(a)). Unremarkable reactive secondary lymphoid follicles were seen adjacent to the nodular area. The nodules contained scattered large atypical cells in a background of small lymphocytes and occasional histiocytes (Figure 2(b)). The large cells showed features characteristic of lymphocyte predominant (LP) cells, including multilobated nuclei with watery chromatin, single central nucleoli, and sparse cytoplasm.

**Figure 3:**
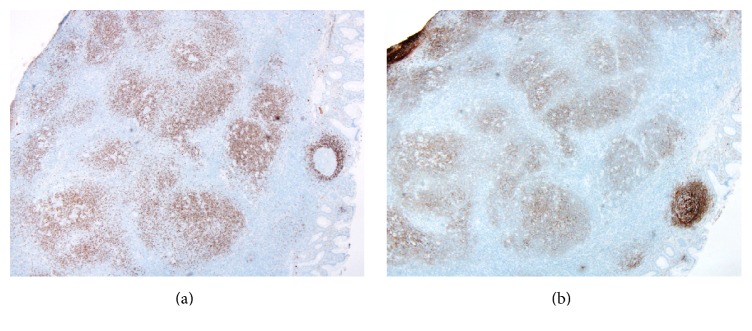
Immunoarchitecture of the ileal lesion. (a) Low-power image of an IgD immunohistochemical stain shows numerous positive cells within the nodules. The mantle zone of an adjacent reactive follicle is also seen (right). (b) Low-power image of a CD21 stain shows expanded follicular dendritic cell meshworks associated with the nodules.

**Figure 4:**
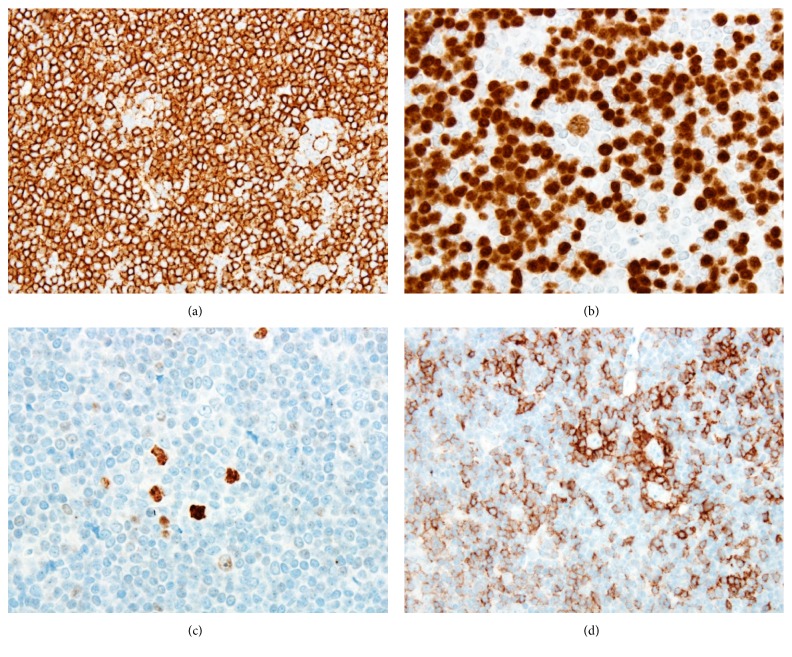
Immunohistochemical features of the neoplastic cells. (a) High-power image of a CD20 immunohistochemical stain shows expression by the scattered large neoplastic cells. Numerous small B-cells in the background also are positive. (b) A PAX5 stain also shows positivity in the large neoplastic cells and small background B-cells. (c) An OCT2 stain shows strong nuclear staining in the neoplastic cells. (d) A PD-1 stain shows rosetting of the large neoplastic cells by small PD-1-positive T lymphocytes.

At low power, immunohistochemical stains showed that the nodules contained mostly CD20-positive B-cells that coexpressed IgD and were associated with expanded CD21-positive follicular dendritic cell meshworks (Figure 3). At higher power, the large atypical cells were observed to express CD45, CD20 (Figure 4(a)), PAX5 (Figure 4(b)), OCT2 (Figure 4(c)), and BCL6. Kappa and lambda stains showed them to be kappa light chain-restricted. They were negative for CD30, CD15, and EMA. The large cells were rosetted by CD3-positive and PD-1-positive T-cells (Figure 4(d)). These immunophenotypic findings confirmed the diagnosis of NLPHL.

There was no evidence of disease at other sites (clinical stage IEA). The patient had a complete response to chemotherapy with rituximab, cyclophosphamide, doxorubicin, vincristine, and prednisone (R-CHOP). Colonoscopy performed 24 months after diagnosis was normal, and imaging studies performed 30 months after diagnosis revealed no evidence of disease.

## 3. Discussion

NLPHL is a rare clonal B-cell neoplasm that typically presents with localized peripheral lymphadenopathy of longstanding duration [1]. It shares with the more common classical form of Hodgkin lymphoma certain clinical presentations and morphologic characteristics but has a distinct immunophenotype and molecular pathogenesis. Extranodal NLPHL is rare. The liver and spleen may be involved in the presence of advanced stage nodal disease; bone marrow involvement is significantly less common. Siebert et al. have described 13 cases of NLPHL with extranodal involvement of Waldeyer's ring, spleen, liver, and bone marrow, all of whom also had nodal disease [2]. Chang et al. have reported on 51 NLPHL specimens from 16 patients and found a similar spectrum of extranodal sites, also generally accompanying nodal disease, and identified the salivary gland as an additional extranodal site [3]. Data from the European Task Force on Lymphoma found a low incidence of extranodal organ involvement by NLPHL, including liver (3%), bone marrow (1%), lung (1%), skeleton (1%), and other organs (2%) [4]. Parikh et al. have reported a case of NLPHL involving the pancreas [5], but true gastrointestinal tract involvement is exceedingly rare. Bohn-Sarmiento et al. have reported a case of primary NLPHL involving the appendix in a 62-year-old male who presented with acute appendicitis [6]. More recently, Bagwan et al. have described a 32-year-old male who was being evaluated for axillary lymphadenopathy when he presented with an acute abdomen and was found to have small bowel perforation associated with NLPHL in addition to a T-cell/histiocyte-rich large B-cell lymphoma-like component [7]. Both components also were present in an axillary lymph node biopsy specimen. The case we present here is unique in its incidental discovery during screening colonoscopy in an asymptomatic patient.

Identification of the key morphologic features of NLPHL on H&E stains is essential to prompt a comprehensive panel of confirmatory immunostains leading to correct diagnosis. In the present case, the morphologic and immunophenotypic features were quite characteristic despite the unusual anatomic site. The morphologic features included the typical nodular architecture, the cytological features of the neoplastic cells including nuclear multilobation leading to the designation “popcorn” cells, and the background of reactive small lymphocytes and bland histiocytes. The architectural features in this case characterize the “classic” pattern of NLPHL, but variant histologic patterns also exist [8]. NLPHL must be distinguished from reactive conditions, including progressive transformation of germinal centers (PTGC) [9]. In excisional lymph node biopsies, effacement of the nodal architecture is useful to prompt a careful search for the large neoplastic cells, but architectural clues are often absent in needle cores, other small biopsies, and extranodal anatomic sites. Other important considerations in the differential diagnosis of NLPHL include non-Hodgkin lymphomas such as low-grade B-cell lymphomas or occasionally T-cell lymphoma and classical Hodgkin lymphoma (CHL) [1]. Immunohistochemistry is critical in these distinctions, particularly in the differentiation between NLPHL and the lymphocyte-rich subtype of CHL. As in the case presented here, NLPHL expresses a complete mature B-cell phenotype, including generally strong immunohistochemical staining for surface B-cell markers such as CD20, immunoglobulins, and B-cell transcription factors such as OCT2. In contrast, NLPHL typically lacks staining for CD30 and CD15, which are present in most cases of CHL. Of note, it is not always possible to make a definitive distinction between NLPHL and lymphocyte-rich CHL, and the existence of cases with intermediate features is recognized [10].

In summary, we present a unique case of diagnosis of NLPHL of the ileum as an incidental finding on routine colonoscopy. This case highlights the importance of awareness of the spectrum of clinical presentations, careful morphologic evaluation of any atypical lymphoid process, and use of a comprehensive panel of immunohistochemical stains for the correct diagnosis of NLPHL and other lymphomas in unusual anatomic sites.
